# Patient Preferences For Specialty Pharmacy Services: A Stated Preference Discrete-Choice Experiment in China

**DOI:** 10.3389/fpubh.2020.597389

**Published:** 2020-12-09

**Authors:** Qinyuan Hu, Haiyao Hu, Ming Hu, Yumei Yang, Zhiang Wu, Naitong Zhou

**Affiliations:** ^1^West China School of Pharmacy, Sichuan University, Chengdu, Sichuan, China; ^2^West China School of Medicine, Sichuan University, Chengdu, Sichuan, China; ^3^Yeehong Business School, Shenyang Pharmaceutical University, Beijing, China

**Keywords:** specialty pharmacy, patient preference, pharmacy service, DCE, conjoint analysis (CA)

## Abstract

**Objectives:** To survey, analyze, and ascertain the preferences for specialty pharmacy services among patients requiring complex care and to provide evidence to support specialty pharmacy service decision-making in China.

**Methods:** To identify essential service attributes and levels, a review of the literature, discussions with specialty pharmacy managers and a pilot questionnaire were conducted. A D-efficient fractional factorial design was used to generate the discrete-choice experiment (DCE) questionnaire. A face-to-face survey of patients with chronic illness and their families or friends was conducted at three specialty pharmacies in Chengdu and Qingdao, China. A mixed logit model was used for estimation.

**Results:** Six relevant attributes were identified and incorporated into the DCE questionnaire. A total of 417 participants completed the survey (mean age 43 years, 45.1% males), and 32.1% had lung cancer. The conditional relative importance showed that the most critical attribute was “frequency of telephone follow-up to monitor adverse drug reactions (ADRs), “followed by “mode of drug delivery,” “provider of medication guidance services,” and “availability of medical insurance consultation”; the least important attribute was “business hours.” A 1 min increase in time spent led to a 0.73% decrease in the probability that a service profile would be chosen. Negative preferences were noted for ADR monitoring by telephone follow-up once a year (β = −0.23, *p* < 0.001) and business hours [8:30–20:00 (Monday to Friday), 8:30–17:30 (weekend)] (β = −0.12, *p* < 0.001). Compared with women, men had a higher preference for service monitoring ADRs once every 3 months.

**Conclusions:** Preference measurements showed that “frequency of telephone follow-up to monitor ADRs” had the most critical impact on decisions, followed by “mode of drug delivery.” Specialty pharmacies in China need to take these findings into account to improve their design to increase uptake and patient loyalty.

## Introduction

Specialty pharmacies, which originated in the United States in the 1980s, began to grow as hospital costs soared, and many patients opted for out-of-hospital treatment (1). China's specialty pharmacies emerged in 2002. In China, specialty pharmacies, also known as direct-to-patient (DTP) pharmacies, are a special type of retail pharmacy that deals with drugs for special diseases that require professional and personalized drug treatment management services (2). In China, a series of policies have forced medical institutions to reduce drug sales, especially for high-cost drugs: the hierarchical diagnosis and treatment system (2015) (3), regulations that prohibit medical institutions from limiting the outflow of prescriptions (2016) (4), zero price addition (2017) (5), and centralized procurement in significant quantities by public health care institutions to reform the drug procurement system and make medicines more affordable for patients (2019) (6). In this context, for chronic patients, specialty pharmacies is playing an increasingly important role in meeting the purchase demand for prescription drugs.

Additionally, specialty pharmacies are also taking on roles in chronic disease management. When patients purchase drugs from specialty pharmacies, they also need personalized medication advice and professional distribution services through licensed pharmacists. In 2019, the scale of drug retail in China was 1795.5 billion yuan, and the growth rate slowed. However, the scale of specialty pharmacies has been accelerating in the past 2 years. In 2019, there were ~1,280 specialty pharmacies, with a sale volume of more than 10 billion yuan. It is estimated that the sales volume will reach 610 billion yuan in 2020 (7).

Pharmaceutical care (patient education, follow-up, etc.) in specialty pharmacies, which is critical for improving patient compliance and medication effects, is designed from the perspective of pharmaceutical care providers and clinical workers, but the views of patients are lacking.

The stated preference (SP) method is widely used in the health sector to investigate preferences and to evaluate health outcomes. It includes standard gamble, time trade-off, person trade-off, contingent valuation and discrete-choice experiments (DCEs) (8). By eliciting the preferences for and values of goods/services in markets that exist or do not exist, DCEs provide rich data sources for economic evaluation and decision-making (8). DCEs can be used to determine the intensity of medical service preferences, the relative importance of different attributes of services, and the marginal rate of substitution between attributes (9). In recent years, DCEs have been increasingly used to identify and evaluate research on health outcomes, medical services, and even pharmacy services (10).

Additionally, in recent years, there has been an increasing number of studies on the community pharmacy service preference of patients using DCEs (11–15). However, there is no previous research on patients' demand for pharmaceutical services in specialty pharmacies. This study aims to survey, analyze, and ascertain the preferences for specialty pharmacy services of patients requiring complex care to provide evidence to support specialty pharmacy service-related decision-making in China.

## Methods

### Selection of Attributes and Levels

To select attributes for this study, a four-step approach was used. First, the terms “patient preference,” “consumer preference,” “community pharmacy,” “specialty pharmacy,” “pharmacy service,” and “discrete choice experiment” were used to search the PubMed and initially extract attributes that may affect patients' choice preference. Among the discrete choice studies published in the field of health economics from 2013 to 2017, most analyzed the impact of 4–9 attributes (16). In the DCE studies on pharmacy services published between 2006 and 2015, the most commonly used attributes were waiting time, service provider, business hours, price, professional guidance, consultation time, and so on (17). In the research on pharmacy quality evaluation (18–21), common evaluation indicators include drug price, pharmacy staff's service attitude, whether to provide drug consulting services, waiting time, whether to provide delivery services, etc. Other evaluation indicators include pharmacy location and transportation convenience, whether to provide health management service, pharmacy layout, etc.

A list was created based on a combination of China's existing advanced specialty pharmacy experience (22, 23) and the results of the literature review. This list consisted of five attributes: the provision of medication guidance services, mode of drug delivery, business hours, frequency of telephone follow-up to monitor adverse drug reactions (ADRs), and availability of reimbursement.

Then, the list was discussed with relevant experts and specialty pharmacy managers to confirm its validity. As a result, two more attributes were added: the availability of medical insurance consultation and the average waiting time for purchasing.

Then, a pilot test was conducted to determine the importance of each criterion relative to the others, and another round of discussion with experts was conducted based on the pilot results. Finally, the availability of reimbursement was moved to the essential information part for respondents who focused on this attribute and ignored the others, which could bias the model estimation (24).

The cost was not included in this study because services at specialty pharmacies are free to offset the high drug costs. The selection of levels per attribute was based on considerations of the actual situation in China. As recommended by the International Society for Pharmacoeconomics and Outcomes Research (ISPOR) (24), each attribute should have no more than three or four levels, and should not contain limits to affect the results. The selected attributes and levels are shown in Table 1.

**Table 1:** Attributes and levels.

**Attributes**	**Levels**
Provision of medication guidance services	A doctor (online) and a pharmacist (at the pharmacy)
	A doctor (periodically offline) and a pharmacist (at the pharmacy)
	Only a pharmacist (at the pharmacy)
	None
Mode of drug delivery	To patients' homes (citywide)
	To designated hospitals
	To patients' homes (only in the central districts of cities)
	None
Business hours	24 h per day
	8:30–20:00 (Monday to Friday), 8:30–17:30 (weekends)
	8:30–17:30 every day
	8:30–17:30 (Monday to Friday); weekends off
Frequency of telephone follow-up to monitor ADRs	Once every 3 months
	Once every 6 months
	Once a year
	None
Availability of medical insurance consultation	Yes
	No
Average waiting time for purchasing	10 min
	30 min
	50 min
	70 min

### Experimental Design

A D-efficient fractional factorial experimental design, the most commonly used metric, was employed to produce near-maximum D-efficiency and maximum precision using SAS 9.4 (SAS Institute, Inc., Cary, NC, USA) (24–27). The design contained 16 choice tasks, each involving two scenarios. The number of tasks was obtained from Sawtooth software and corresponded to the recommended number (24). Each scenario described a combination of specialty pharmacy service attributes and levels. Respondents had to select one of the two alternative scenarios in each choice set. A dominance test resulted from the generation of the choice sets as part of the experimental design in which one alternative had better levels of services than the other alternative. There are two reasons not to delete this part of the data: (1) deleting responses might result in the removal of valid preferences and sample selection bias and reduce the statistical efficiency, external validity and power of the estimated choice models; (2) evidence suggests that random utility theory might be able to cope with such preferences because a substantial portion of the error variance consists of unobserved taste heterogeneity across respondents (28–30). Therefore, we did not delete the data in which the dominant alternative was not chosen. We also collected demographic-related pharmacy consumption information to identify respondent characteristics and to perform subgroup analyses (31).

### Study Population

The sample size of 450 respondents exceeded the sample size recommended by Orme for subgroup analysis (24, 26). To ensure that the respondents understood our questionnaire and to reach older populations (27) better, face-to-face and paper-based surveys of patients were conducted at three specialty pharmacies in Chengdu and Qingdao, China. To ensure the representativeness of the research results, we choose Qingdao and Chengdu, the first cities to set up specialty pharmacies, as the research sites. We selected one specialty pharmacies in each city that was listed among China's top 10 specialty pharmacies in 2019, chosen by the China Pharmaceutical Material Association, as the sample pharmacy for the survey. The third pharmacy is also located in Chengdu and has increased in scale and sales volume in recent years. The three sample pharmacies are representative enough in terms of business scale, sales volume, pharmacist level and disease diversity among the service's customers (32). We employed undergraduate pharmacy majors to lead the surveys. Participants were eligible if they were over 18 years old, did not have conditions that involved cognitive deficits or severe hearing and visual impairment, and spoke Chinese. We did not include participants who were using a specialty pharmacy for the first time because we thought that they would not have sufficient preferences regarding this kind of pharmacy. To motivate the participants in our surveys, we told them that the study aimed to improve the services of pharmacies and the outcomes of medication intervention, and we also provided a gift as a token of gratitude.

### Data Analysis

We used evenly spaced attribute levels to interpret the estimated effects of the average waiting time for purchasing (10, 30, 50, and 70 min) (8). To test whether the average waiting time for purchasing was appropriate as a continuous variable, we examined the results of a model in which the numeric levels were treated as categorical (33). We set this variable as a continuous variable for two reasons. First, the results of the categorical models showed that the relative marginal utility of a one-unit change in the waiting time measure was equal regardless of whether the variable was treated as continuous or categorical. Specifically, the coefficient differences of the four levels of this attribute were 0.27, 0.37, and 0.38, and the ratio of the differences corresponding to the two adjacent levels was relatively close [0.27/(30–10) ≈ 0.37/(50–30) ≈ 0.38/(70–50)]. Second, the continuous model had better goodness of fit with a lower Bayesian information criterion (BIC) value than the categorical models (34). We set the other attribute levels as categorical and uniformly distributed to obtain information about the respondents' willingness to spend and the trade-offs among these factors (35). Because effects coding has desirable properties for modeling conjoint-analysis data (24), this study used effects coding to specify the categorical attribute levels.

A random-parameters logit (RPL) regression model was used to analyze the choice data collected in this DCE; this model allowed for unobserved or random preference variation and could incorporate the cross-sectional panel structure of the data (24). The model was estimated using the mixlogit command in Stata 15 (StataCorp, College Station, TX). All variables, except constants, were set as random. The mean value and standard deviation appeared in the model estimation results. Finally, parameters for which the standard deviation had no statistical significance (*P* > 0.05) were set as fixed coefficients (36), and parameters for which the standard deviation was statistically significant were set as random coefficients.

Subgroup analyses examined whether preferences differed systematically based on patient characteristics and disease varieties. A conditional logit was used to construct the model. Fisher's permutation test was conducted to compare the coefficients between groups using the bdiff command in Stata (33, 37, 38). The analyses excluded participants who did not complete all choice tasks. The lowest level of each attribute, which we expected to be the worst, was used as the omitted level.

In addition to preference weight, we also calculated the conditional relative importance, time trade-off, and choice probability (35). The conditional relative importance represented the maximum change in utility achievable with any attribute. It was calculated as the difference between the highest preference weight and the lowest preference weight for the same attribute (39). The time trade-off allowed us to estimate how much time a respondent would be willing to give up to experience an improvement in an attribute. It was calculated as the ratio of the value of the coefficient to the negative of the time attribute (35). The probability of choosing a given service change as the attribute levels change (35).

## Results

### Sample Characteristics

A total of 417 participants completed the survey. The characteristics of the sample, which included 315 patients in Chengdu and 102 in Qingdao, are presented in Table 2. The mean age was 43 years. One hundred twenty-six respondents had lung cancer, while the others (267 respondents) had leukemia (5.0%), hepatitis C (4.1%), liver cancer (3.8%), breast cancer (3.8%), and other diseases. A total of 81.2% of the participants' medicines were reimbursable. Twenty-five (6%) respondents did not choose the dominant scenario in the dominant test task.

**Table 2:** Demographic and clinical characteristics.

**Characteristic**	**Subjects (*N* = 393)**
Age (y), mean (range)	43 (19–82)
**Gender**, ***n*** **(%)**	
Male	191 (45.8%)
Female	226 (54.2%)
**Ethnicity**, ***n*** **(%)**	
Han	404 (96.9%)
Minority	13 (3.1%)
**Place of registration**, ***n*** **(%)**	
Rural	154 (36.9%)
Urban	262 (62.8%)
**Marital status**, ***n*** **(%)**	
Married	326 (78.2%)
Single	59 (14.1%)
Divorced	21 (5.0%)
Widowed	10 (2.4%)
**Working status**, ***n*** **(%)**	
Employed	242 (58.0%)
Retired	80 (19.2%)
In school	10 (2.4%)
Unemployed	85 (20.4%)
**Education**, ***n*** **(%)**	
Primary school or below	19 (4.6%)
Middle school	86 (20.6%)
High school	89 (21.3%)
Bachelor's degree or higher	222 (53.2%)
**Monthly income of each family member**, ***n*** **(%)**	
Below 2,000 yuan/month	53 (12.7%)
2,000–4,999 yuan/month	145 (34.8%)
5,000–9,999 yuan/month	140 (33.6%)
10,000 yuan/month and above	74 (17.7%)
**Type of participant**, ***n*** **(%)**	
Patient	152 (36.5%)
Patient's family member	246 (59.0%)
Patient's friend	18 (4.3%)
**Type of medical insurance**, ***n*** **(%)**	
Basic medical insurance system for urban workers	216 (51.8%)
Urban and rural resident basic medical insurance	147 (35.3%)
Commercial health insurance	51 (12.2%)
None	2 (0.5%)
**Type of disease**, ***n*** **(%)**	
Lung cancer	134 (32.1%)
Leukemia	21 (5.0%)
Hepatitis C	17 (4.1%)
Breast cancer	16 (3.8%)
Liver cancer	16 (3.8%)
Ankylosing spondylitis	15 (3.6%)
Rheumatoid arthritis	15 (3.6%)
Others	142 (44.0%)
Mean duration of illness (y), mean (max)	4 (35)
**Number of visits to this pharmacy in the past year**, ***n*** **(%)**	
<5	187 (44.8%)
5–9	98 (23.5%)
10–14	98 (23.5%)
≥15	30 (7.2%)
**Average cost per visit**, ***n*** **(%)**	
Less than 99	5 (1.2%)
100–499	18 (4.3%)
500–1,999	101 (24.2%)
2,000–9,999	224 (53.7%)
Over 10,000	53 (12.7%)
**Availability of reimbursement for drugs**, ***n*** **(%)**	
Yes	341 (81.8%)
No	53 (12.7%)
Do not know	21 (5.0%)

### Random Parameter Model

The results of the mixed logit model are presented in Table 3. The coefficient of the reference level was obtained by calculating the negative of the sum of the estimated preference weights for all non-omitted levels of the attribute (34). Telephone follow-up to monitor ADRs once every 3 months had the highest preference weight (0.53), followed by drug delivery to patients' homes (citywide) (0.49). The negative preferences were for ADR monitoring by telephone follow-up once a year (β = −0.23, *p* < 0.001) and business hours [8:30–20:00 (Monday to Friday), 8:30–17:30 (weekend)] (β = −0.12, *p* < 0.001). Only the level of “drug delivery to designated hospitals” was not statistically significant. Participants were willing to spend an extra 36 min to receive telephone follow-up to monitor ADRs once every 3 months.

**Table 3:** Results of the mixed logit model.

**Attribute**	**β**	**Standard deviation (SD)**	**Trade-off (minutes)**	**Choice probability**	**Conditional relative importance**	**Rank**
Provision of medication guidance services					0.88	3
A doctor (online) and a pharmacist (at the pharmacy)	0.20^***^	0.32	14	37.23%^***^		
A doctor (periodically offline) and a pharmacist (at the pharmacy)	0.30^***^	0.52	20	41.37%^***^		
Only a pharmacist (at the pharmacy)	0.09^*^	−0.07	6	32.41%^***^		
None	−0.58		Reference	Reference		
Mode of drug delivery					1.10	2
To patients' homes (citywide)	0.49^***^	0.53	33	50.07%^***^		
To designated hospitals	−0.02	0.09	−1	28.64%^***^		
To patients' homes (only in the central districts of cities)	0.14^**^	0.33	10	36.04%^***^		
None	−0.61		Reference	Reference		
Business hours					0.26	5
24 h per day	0.12^**^	0.15	8	12.47%^***^		
8:30–20:00 (Monday to Friday), 8:30–17:30 (weekends)	−0.12^**^	0.12	−9	0.23%		
8:30–17:30 every day	0.13^***^	−0.08	9	13.06%^***^		
8:30–17:30 (Monday to Friday), weekends off	−0.13		Reference	Reference		
Frequency of telephone follow-up to monitor ADRs					1.21	1
Once every 3 months	0.53^***^	0.56	36	53.92%^***^		
Once every 6 months	0.37^***^	−0.12	26	48.23%^***^		
Once a year	−0.23^***^	−0.09	−16	22.13%^***^		
None	−0.68		Reference	Reference		
Availability of medical insurance consultation					0.71	4
Yes	0.35^***^	0.51	24	33.95%^***^		
No	−0.35		Reference	Reference		
Average waiting time for purchasing	−0.01^***^			−0.73%^***^		

*** *P < 0.001*,

** *P < 0.01*,

* *P < 0.05*.

The conditional relative importance analysis revealed that the participants valued the frequency of telephone follow-up to monitor ADRs the most (1.21), followed the mode of drug delivery (1.10). The provision of medication guidance and availability of medical insurance consultation services were the third (0.88) and fourth most important attributes (0.71), respectively. Business hours were the least important attribute (0.26). Compared to the availability of an online doctor and a pharmacist at the pharmacy (0.20), the participants preferred to have a doctor periodically available offline and a pharmacist available at the pharmacy (0.30).

The probability that a service profile would be chosen from a profile pair was 53.92% higher if the service included monitoring ADRs once every 3 months than if it did not monitor ADRs. Also, adding a semiannual call to monitor ADRs led to a 48.23% increase. The service choice probability increased by 50.07% if the service offered drug delivery to patients' homes (citywide) as opposed to no delivery. A 1 min increase in time spent led to a 0.73% decrease in the probability that a service profile would be chosen.

### Subgroup Analysis

No significant differences in the preference estimates were shown between people who had lung cancer and those who had other diseases. Compared with women, men had a greater preference for ADR monitoring service once every 3 months. Compared to rural participants, urban participants cared more about ADR monitoring service once every 3 months and medical insurance consultation. Employed individuals valued consultation regarding medical expenses less than other individuals did (including retired individuals, individuals in school and unemployed individuals). Those with a monthly income per family member below 5,000 yuan showed a greater preference for medical insurance consultation (see Appendix A in the Supplementary Material).

## Discussion

This study investigated patient preferences for pharmaceutical services at specialty pharmacies in China. The importance of these service attributes revealed to some extent their value to society, which is in line with patient requirements and consistent with the themes of the patient-centered era. Besides, pharmacy services that are better aligned with patient expectations can improve patients' attraction and loyalty to pharmacies (40). Studies have shown a certain link between pharmacy loyalty and persistence with treatment (41, 42). Therefore, improving pharmacy services can also enhance persistence with treatment to some extent. The results show that the frequency of telephone follow-up to monitor ADRs was the most crucial attribute to those included in this study, followed by the provision of medication guidance services, mode of drug delivery, business hours, and availability of medical insurance consultation.

Telephone follow-up to monitor ADRs was the most important attribute, which is not surprising for two major reasons. First, most patients who use specialty pharmacies have more severe, longer-lasting illnesses than other patients. Due to their use of numerous and multiple drugs with high toxicity, these patients are more likely to experience ADRs (43). However, most patients find drug instructions, both technical and lengthy, and doctors cannot summarize many ADRs. Therefore, patients have an urgent need for follow-up to monitor ADRs, and telephone follow-up provided by specialty pharmacies can well meet this need. Second, telephone follow-up can save time and effort, especially for patients with severe illnesses, e.g., cancer, and it can also protect patient privacy. As the results show, the higher the frequency of telephone follow-up, the greater the patient preference; the 3 month follow-up had the highest preference weight, while the patients did not accept the 1 year follow-up.

Drug delivery also obtained high importance. A good drug distribution model, i.e., delivering drugs to patients' homes, can not only better ensure the quality of drugs, especially cold-chain drugs, but can also improve the availability of drugs to a certain extent. Such a model can ensure that the patient receives refills as soon as the previous prescription runs out, which is crucial for patients with severe and chronic diseases. This result is in line with that of earlier work from Australia, which focused on community pharmacy services for managing chronic conditions (12). Inevitably, however, unrestricted distribution means higher costs for pharmacies, which they must consider at their discretion.

Regarding the provision of medication guidance services, patients prefer the combined availability of a doctor and a pharmacist rather than guidance from just a pharmacist. The reason may be that Chinese pharmacists do not yet have professional expertise equal to that of doctors. This finding is similar to that of another report regarding consumer preferences for a medication review service for elderly patients in England (10). Compared with online guidance from doctors and offline advice from pharmacists, patients are more likely to choose face-to-face guidance from doctors and pharmacists. Such guidance can be provided by regularly bringing doctors to pharmacies, conducting regular patient education activities, or having pharmacies organize patient trips to hospitals for free consultations.

The significant and negative coefficient of the average waiting time indicates that it is also a decisive factor affecting patient preferences. The longer the wait is, the more negative feelings the patient experiences. Service counters and staffing that is appropriate for the number of patients can address this problem to some extent. Furthermore, an efficient management mechanism can also reduce patients' waiting time, for example, by setting pharmacy visit times for specific disease types and large-scale scheduling activities, such as charity drug collections, for times when few people are waiting. In addition, an excellent waiting area environment can reduce patients' negative feelings, and pharmacies can consider having a waiting area equipped with comfortable seats, drinking water, etc.

Subgroup analyses enable decision-makers to provide accurate and personalized services to different types of patients while maximizing the impact of limited resources. Our results show that urban participants place greater value on telephone follow-up services, possibly because people living in cities are exposed to more novelties than people living in rural areas, making them more willing to agree to ADR monitoring by telephone. Employed individuals have a stable income; thus, they are less focused on consultations regarding health care spending. The fact that those with a monthly income per family member of more than 5,000 yuan care about medical insurance consultation more than others can also be interpreted in this manner.

Our study has several limitations. First, our sample may not represent a wide range of specialty pharmacy patients. The preferences of specialty pharmacy patients in Chengdu and Qingdao may be different from those of patients throughout China. Second, the service scheme that we designed using the SP method may deviate from the actual service scheme, which is a common limitation in DCE studies (39). Third, repeated selection tasks may cause participants to lose patience and develop cognitive fatigue, leading to errors in the results (39). Fourth, because our choice sets included a dominant task, the error term was decreased (28). Fifth, the study did not address economic attributes, i.e., cost, because specialty pharmacies in China typically provide high-cost drugs at prices high enough to cover the cost of services.

## Conclusions

Patients in specialty pharmacies showed a maximum preference for the frequency of telephone follow-up to monitor ADRs, followed by the mode of drug delivery. Our results also found differences in service preferences among patients with different characteristics. Specialty pharmacies in China need to take these findings into account to improve their design to increase uptake and patient loyalty.

## Data Availability Statement

The original contributions presented in the study are included in the article/Supplementary Material, further inquiries can be directed to the corresponding author/s.

## Ethics Statement

The studies involving human participants were reviewed and approved by Medical Ethics Committee of Sichuan University. The patients/participants provided their written informed consent to participate in this study.

## Author Contributions

QH: drafting the manuscript. HH and YY: revising the manuscript critically for important intellectual content. All authors contributed to the article and approved the submitted version.

## Conflict of Interest

The authors declare that the research was conducted in the absence of any commercial or financial relationships that could be construed as a potential conflict of interest.
