# Supplementary material for: Patient Preferences For Specialty Pharmacy Services: A Stated Preference Discrete-Choice Experiment in China
Source: Front Public Health. 2020 Dec 9;8:597389. doi: 10.3389/fpubh.2020.597389 (PMC7755860; doi:10.3389/fpubh.2020.597389)
Supplement: Supplementary file 1 [file Table_1.DOCX]

## Appendix A – Stratified conditional logit analyses

# Table 1 Preference estimates for stratified sample based on gender

| **Attribute** | **Level** | **β_0_-β_1_** | **Frequency** | **P-value** |
| --- | --- | --- | --- | --- |
| Average waiting time for purchasing |  | 0.001 | 33 | 0.33 |
|  | Doctor (online) and pharmacist (at the pharmacy) | 0.062 | 18 | 0.18 |
|  | Doctor (periodically offline) and pharmacist (at the pharmacy) | 0.07 | 23 | 0.23 |
|  | Only pharmacist (at the pharmacy) | -0.131 | 92 | 0.08 |
| Mode of drug delivery | To patients' homes (city-wide) | 0.026 | 40 | 0.4 |
|  | To designated hospitals | 0.034 | 27 | 0.27 |
|  | To patients' homes (only the central districts of the city) | 0.047 | 31 | 0.31 |
| Business time | 24 hours per day | 0.015 | 52 | 0.48 |
|  | 8:30-20:00 (Monday to Friday), 8:30-17:30 (weekend) | -0.063 | 79 | 0.21 |
|  | 8:30-17:30, every day | 0.074 | 20 | 0.2 |
| Frequency of telephone follow-up to monitor ADR | Once every three months | 0.145 | 3 | 0.03 |
|  | Once half a year | -0.026 | 64 | 0.36 |
|  | once a year | -0.021 | 58 | 0.42 |
| Availability of medical insurance consultation | Yes | 0.017 | 33 | 0.33 |

*Notes:* β_1_ refers to the coefficient of male; β_0_ refers to the coefficient of female.

# Table 2 Preference estimates for stratified sample based on resident

| **Attribute** | **Level** | **β_0_-β_1_** | **Frequency** | **P-value** |
| --- | --- | --- | --- | --- |
| Average waiting time for purchasing |  | 0.002 | 15 | 0.15 |
|  | Doctor (online) and pharmacist (at the pharmacy) | -0.024 | 61 | 0.39 |
|  | Doctor (periodically offline) and pharmacist (at the pharmacy) | 0.054 | 21 | 0.21 |
|  | Only pharmacist (at the pharmacy) | 0.022 | 39 | 0.39 |
| Mode of drug delivery | To patients' homes (city-wide) | 0.009 | 42 | 0.42 |
|  | To designated hospitals | -0.096 | 96 | 0.04 |
|  | To patients' homes (only the central districts of the city) | 0.113 | 5 | 0.05 |
| Business time | 24 hours per day | 0.059 | 20 | 0.2 |
|  | 8:30-20:00 (Monday to Friday), 8:30-17:30 (weekend) | -0.062 | 92 | 0.08 |
|  | 8:30-17:30, every day | 0.002 | 54 | 0.46 |
| Frequency of telephone follow-up to monitor ADR | Once every three months | -0.091 | 99 | 0.01 |
|  | Once half a year | 0.084 | 9 | 0.09 |
|  | once a year | 0.011 | 43 | 0.43 |
| Availability of medical insurance consultation | Yes | -0.069 | 100 | 0 |

*Notes:* β_1_ refers to the coefficient of the rural; β_0_ refers to the coefficient of the urban.

# Table 3 Preference estimates for stratified sample based on marital status

| **Attribute** | **Level** | **β_0_-β_1_** | **Frequency** | **P-value** |
| --- | --- | --- | --- | --- |
| Average waiting time for purchasing |  | -0.002 | 55 | 0.45 |
|  | Doctor (online) and pharmacist (at the pharmacy) | -0.031 | 49 | 0.49 |
|  | Doctor (periodically offline) and pharmacist (at the pharmacy) | 0.1 | 31 | 0.31 |
|  | Only pharmacist (at the pharmacy) | 0.034 | 43 | 0.43 |
| Mode of drug delivery | To patients' homes (city-wide) | 0.267 | 26 | 0.26 |
|  | To designated hospitals | -0.014 | 52 | 0.48 |
|  | To patients' homes (only the central districts of the city) | -0.165 | 77 | 0.23 |
| Business time | 24 hours per day | 0.148 | 36 | 0.36 |
|  | 8:30-20:00 (Monday to Friday), 8:30-17:30 (weekend) | -0.089 | 59 | 0.41 |
|  | 8:30-17:30, every day | -0.008 | 62 | 0.38 |
| Frequency of telephone follow-up to monitor ADR | Once every three months | 0.104 | 31 | 0.31 |
|  | Once half a year | 0.029 | 53 | 0.47 |
|  | once a year | -0.075 | 48 | 0.48 |
| Availability of medical insurance consultation | Yes | -0.026 | 77 | 0.23 |

*Notes:* β_1_ refers to the coefficient of the married; β_0_ refers to the coefficient of the others.

# Table 4 Preference estimates for stratified sample based on working status

| **Attribute** | **Level** | **β_0_-β_1_** | **Frequency** | **P-value** |
| --- | --- | --- | --- | --- |
| Average waiting time for purchasing |  | 0.002 | 21 | 0.21 |
|  | Doctor (online) and pharmacist (at the pharmacy) | 0.042 | 29 | 0.29 |
|  | Doctor (periodically offline) and pharmacist (at the pharmacy) | 0.045 | 24 | 0.24 |
|  | Only pharmacist (at the pharmacy) | -0.047 | 74 | 0.26 |
| Mode of drug delivery | To patients' homes (city-wide) | -0.041 | 63 | 0.37 |
|  | To designated hospitals | 0.001 | 52 | 0.48 |
|  | To patients' homes (only the central districts of the city) | 0.021 | 42 | 0.42 |
| Business time | 24 hours per day | -0.076 | 76 | 0.24 |
|  | 8:30-20:00 (Monday to Friday), 8:30-17:30 (weekend) | 0.059 | 24 | 0.24 |
|  | 8:30-17:30, every day | -0.055 | 71 | 0.29 |
| Frequency of telephone follow-up to monitor ADR | Once every three months | -0.069 | 94 | 0.06 |
|  | Once half a year | 0.035 | 35 | 0.35 |
|  | once a year | 0.016 | 43 | 0.43 |
| Availability of medical insurance consultation | Yes | 0.081 | 3 | 0.03 |

*Notes:* β_1_ refers to the coefficient of the employed; β_0_ refers to the coefficient of the others.

# Table 5 Preference estimates for stratified sample based on education

| **Attribute** | **Level** | **β_0_-β_1_** | **Frequency** | **P-value** |
| --- | --- | --- | --- | --- |
| Average waiting time for purchasing |  | 0.005 | 6 | 0.06 |
|  | Doctor (online) and pharmacist (at the pharmacy) | -0.105 | 78 | 0.22 |
|  | Doctor (periodically offline) and pharmacist (at the pharmacy) | -0.052 | 75 | 0.25 |
|  | Only pharmacist (at the pharmacy) | 0.001 | 56 | 0.44 |
| Mode of drug delivery | To patients' homes (city-wide) | -0.167 | 94 | 0.06 |
|  | To designated hospitals | 0.023 | 34 | 0.34 |
|  | To patients' homes (only the central districts of the city) | 0.019 | 37 | 0.37 |
| Business time | 24 hours per day | -0.161 | 88 | 0.12 |
|  | 8:30-20:00 (Monday to Friday), 8:30-17:30 (weekend) | 0.149 | 13 | 0.13 |
|  | 8:30-17:30, every day | -0.098 | 80 | 0.2 |
| Frequency of telephone follow-up to monitor ADR | Once every three months | 0.052 | 30 | 0.3 |
|  | Once half a year | -0.133 | 87 | 0.13 |
|  | once a year | 0.011 | 38 | 0.38 |
| Availability of medical insurance consultation | Yes | 0.118 | 10 | 0.1 |

*Notes:* β_1_ refers to the coefficient of the educated high school or above; β_0_ refers to the coefficient of the others.

# Table 6 Preference estimates for stratified sample based on each person income of family per month(EPIF)

| **Attribute** | **Level** | **β_0_-β_1_** | **Frequency** | **P-value** |
| --- | --- | --- | --- | --- |
| Average waiting time for purchasing |  | 0.004 | 1 | 0.01 |
|  | Doctor (online) and pharmacist (at the pharmacy) | 0.061 | 22 | 0.22 |
|  | Doctor (periodically offline) and pharmacist (at the pharmacy) | -0.012 | 55 | 0.45 |
|  | Only pharmacist (at the pharmacy) | 0.005 | 64 | 0.36 |
| Mode of drug delivery | To patients' homes (city-wide) | -0.09 | 85 | 0.15 |
|  | To designated hospitals | 0.107 | 7 | 0.07 |
|  | To patients' homes (only the central districts of the city) | -0.078 | 82 | 0.18 |
| Business time | 24 hours per day | -0.053 | 73 | 0.27 |
|  | 8:30-20:00 (Monday to Friday), 8:30-17:30 (weekend) | 0.024 | 43 | 0.43 |
|  | 8:30-17:30, every day | -0.024 | 64 | 0.36 |
| Frequency of telephone follow-up to monitor ADR | Once every three months | 0.013 | 45 | 0.45 |
|  | Once half a year | -0.043 | 71 | 0.29 |
|  | once a year | 0.013 | 43 | 0.43 |
| Availability of medical insurance consultation | Yes | 0.082 | 2 | 0.02 |

*Notes:* β_1_ refers to the coefficient of the sample whose EPIF ＞ 5000 ; β_1_ refers to the coefficient of the others.

# Table 7 Preference estimates for stratified sample based on type of the participants

| **Attribute** | **Level** | **β_0_-β_1_** | **Frequency** | **P-value** |
| --- | --- | --- | --- | --- |
| Average waiting time for purchasing |  | -0.001 | 77 | 0.23 |
|  | Doctor (online) and pharmacist (at the pharmacy) | -0.036 | 71 | 0.29 |
|  | Doctor (periodically offline) and pharmacist (at the pharmacy) | -0.009 | 57 | 0.43 |
|  | Only pharmacist (at the pharmacy) | 0.028 | 36 | 0.36 |
| Mode of drug delivery | To patients' homes (city-wide) | 0.045 | 31 | 0.31 |
|  | To designated hospitals | 0.095 | 9 | 0.09 |
|  | To patients' homes (only the central districts of the city) | -0.017 | 62 | 0.38 |
| Business time | 24 hours per day | 0.02 | 34 | 0.34 |
|  | 8:30-20:00 (Monday to Friday), 8:30-17:30 (weekend) | -0.045 | 79 | 0.21 |
|  | 8:30-17:30, every day | 0.056 | 16 | 0.16 |
| Frequency of telephone follow-up to monitor ADR | Once every three months | 0.074 | 10 | 0.1 |
|  | Once half a year | 0.11 | 6 | 0.06 |
|  | once a year | -0.121 | 95 | 0.05 |
| Availability of medical insurance consultation | Yes | 0.031 | 15 | 0.15 |

*Notes:* β_1_ refers to the coefficient of the patients; β_0_ refers to the coefficient of the others.

# Table 8 Preference estimates for stratified sample based on number of visits to this pharmacy in the past year

| **Attribute** | **Level** | **β_0_-β_1_** | **Frequency** | **P-value** |
| --- | --- | --- | --- | --- |
| Average waiting time for purchasing |  | -0.002 | 81 | 0.19 |
|  | Doctor (online) and pharmacist (at the pharmacy) | -0.049 | 78 | 0.22 |
|  | Doctor (periodically offline) and pharmacist (at the pharmacy) | -0.014 | 51 | 0.49 |
|  | Only pharmacist (at the pharmacy) | 0.044 | 35 | 0.35 |
| Mode of drug delivery | To patients' homes (city-wide) | 0.024 | 37 | 0.37 |
|  | To designated hospitals | -0.153 | 100 | 0 |
|  | To patients' homes (only the central districts of the city) | 0.07 | 18 | 0.18 |
| Business time | 24 hours per day | -0.03 | 63 | 0.37 |
|  | 8:30-20:00 (Monday to Friday), 8:30-17:30 (weekend) | 0.072 | 12 | 0.12 |
|  | 8:30-17:30, every day | -0.094 | 89 | 0.11 |
| Frequency of telephone follow-up to monitor ADR | Once every three months | 0.034 | 29 | 0.29 |
|  | Once half a year | -0.033 | 68 | 0.32 |
|  | once a year | -0.024 | 59 | 0.41 |
| Availability of medical insurance consultation | Yes | -0.078 | 99 | 0.01 |

*Notes:* β_1_ refers to the coefficient of the sample whose number ≤ 5; β_0_ refers to the coefficient of the others.

# Table 9 Preference estimates for stratified sample based on average per consumption

| **Attribute** | **Level** | **β_0_-β_1_** | **Frequency** | **P-value** |
| --- | --- | --- | --- | --- |
| Average waiting time for purchasing |  | 0.001 | 38 | 0.38 |
|  | Doctor (online) and pharmacist (at the pharmacy) | -0.109 | 86 | 0.14 |
|  | Doctor (periodically offline) and pharmacist (at the pharmacy) | 0.124 | 14 | 0.14 |
|  | Only pharmacist (at the pharmacy) | 0.096 | 29 | 0.29 |
| Mode of drug delivery | To patients' homes (city-wide) | 0.16 | 26 | 0.26 |
|  | To designated hospitals | -0.055 | 61 | 0.39 |
|  | To patients' homes (only the central districts of the city) | -0.05 | 51 | 0.49 |
| Business time | 24 hours per day | 0.187 | 18 | 0.18 |
|  | 8:30-20:00 (Monday to Friday), 8:30-17:30 (weekend) | -0.128 | 82 | 0.18 |
|  | 8:30-17:30, every day | -0.04 | 64 | 0.36 |
| Frequency of telephone follow-up to monitor ADR | Once every three months | 0.003 | 50 | 0.5 |
|  | Once half a year | 0.089 | 28 | 0.28 |
|  | once a year | -0.161 | 81 | 0.19 |
| Availability of medical insurance consultation | Yes | -0.039 | 86 | 0.14 |

*Notes:* β_1_ refers to the coefficient of the sample per consumption ≥ 2000; β_0_ refers to the coefficient of the others.

# Table 10 Preference estimates for stratified sample based on whether drugs can be reimbursed

| **Attribute** | **Level** | **β_0_-β_1_** | **Frequency** | **P-value** |
| --- | --- | --- | --- | --- |
| Average waiting time for purchasing |  | 0.001 | 32 | 0.32 |
|  | Doctor (online) and pharmacist (at the pharmacy) | -0.028 | 57 | 0.43 |
|  | Doctor (periodically offline) and pharmacist (at the pharmacy) | 0.035 | 38 | 0.38 |
|  | Only pharmacist (at the pharmacy) | 0.032 | 56 | 0.44 |
| Mode of drug delivery | To patients' homes (city-wide) | 0.085 | 52 | 0.48 |
|  | To designated hospitals | -0.073 | 52 | 0.48 |
|  | To patients' homes (only the central districts of the city) | -0.091 | 63 | 0.37 |
| Business time | 24 hours per day | 0.186 | 35 | 0.35 |
|  | 8:30-20:00 (Monday to Friday), 8:30-17:30 (weekend) | -0.21 | 64 | 0.36 |
|  | 8:30-17:30, every day | 0.046 | 44 | 0.44 |
| Frequency of telephone follow-up to monitor ADR | Once every three months | 0.083 | 33 | 0.33 |
|  | Once half a year | 0.231 | 35 | 0.35 |
|  | once a year | -0.057 | 51 | 0.49 |
| Availability of medical insurance consultation | Yes | 0.048 | 46 | 0.46 |

*Notes:* β_1_ refers to the coefficient of the sample whose medicines could be reimbursed; β_0_ refers to the coefficient of the others.

# Table 11 Preference estimates for stratified sample based on type of [medical](javascript:;) [insurance](javascript:;)

| **Attribute** | **Level** | **β_0_-β_1_** | **Frequency** | **P-value** |
| --- | --- | --- | --- | --- |
| Average waiting time for purchasing |  | -0.002 | 80 | 0.2 |
|  | Doctor (online) and pharmacist (at the pharmacy) | 0.008 | 39 | 0.39 |
|  | Doctor (periodically offline) and pharmacist (at the pharmacy) | -0.04 | 72 | 0.28 |
|  | Only pharmacist (at the pharmacy) | -0.053 | 68 | 0.32 |
| Mode of drug delivery | To patients' homes (city-wide) | -0.028 | 58 | 0.42 |
|  | To designated hospitals | -0.013 | 58 | 0.42 |
|  | To patients' homes (only the central districts of the city) | 0.089 | 25 | 0.25 |
| Business time | 24 hours per day | -0.032 | 61 | 0.39 |
|  | 8:30-20:00 (Monday to Friday), 8:30-17:30 (weekend) | 0.077 | 16 | 0.16 |
|  | 8:30-17:30, every day | -0.094 | 88 | 0.12 |
| Frequency of telephone follow-up to monitor ADR | Once every three months | 0.128 | 8 | 0.08 |
|  | Once half a year | -0.053 | 78 | 0.22 |
|  | once a year | -0.027 | 58 | 0.42 |
| Availability of medical insurance consultation | Yes | 0.094 | 3 | 0.03 |

*Notes:* β_1_ refers to the coefficient of basic medical insurance system for urban workers; β_0_ refers to the coefficient of the sample of urban and rural residents basic medical insurance.

# Table 12 Preference estimates for stratified sample based on pharmacy location

| **Attribute** | **Level** | **β_0_-β_1_** | **Frequency** | **P-value** |
| --- | --- | --- | --- | --- |
| Average waiting time for purchasing |  | -0.003 | 69 | 0.31 |
|  | Doctor (online) and pharmacist (at the pharmacy) | -0.102 | 75 | 0.25 |
|  | Doctor (periodically offline) and pharmacist (at the pharmacy) | 0.057 | 35 | 0.35 |
|  | Only pharmacist (at the pharmacy) | 0.217 | 15 | 0.15 |
| Mode of drug delivery | To patients' homes (city-wide) | 0.298 | 15 | 0.15 |
|  | To designated hospitals | -0.111 | 74 | 0.26 |
|  | To patients' homes (only the central districts of the city) | -0.125 | 75 | 0.25 |
| Business time | 24 hours per day | 0.181 | 20 | 0.2 |
|  | 8:30-20:00 (Monday to Friday), 8:30-17:30 (weekend) | -0.118 | 72 | 0.28 |
|  | 8:30-17:30, every day | -0.096 | 71 | 0.29 |
| Frequency of telephone follow-up to monitor ADR | Once every three months | -0.08 | 75 | 0.25 |
|  | Once half a year | 0.169 | 26 | 0.26 |
|  | once a year | -0.149 | 71 | 0.29 |
| Availability of medical insurance consultation | Yes | -0.05 | 88 | 0.12 |

*Notes:* β_1_ refers to the coefficient of the sample in Chengdu; β_0_ refers to the coefficient of the sample in Qingdao.
